# A Chemical Screen Probing the Relationship between Mitochondrial Content and Cell Size

**DOI:** 10.1371/journal.pone.0033755

**Published:** 2012-03-29

**Authors:** Toshimori Kitami, David J. Logan, Joseph Negri, Thomas Hasaka, Nicola J. Tolliday, Anne E. Carpenter, Bruce M. Spiegelman, Vamsi K. Mootha

**Affiliations:** 1 Broad Institute, Cambridge, Massachusetts, United States of America; 2 Department of Systems Biology, Harvard Medical School, Boston, Massachusetts, United States of America; 3 Department of Molecular Biology, Massachusetts General Hospital, Boston, Massachusetts, United States of America; 4 Department of Cell Biology, Dana-Farber Cancer Institute, Harvard Medical School, Boston, Massachusetts, United States of America; University of Texas Health Science Center at San Antonio, United States of America

## Abstract

The cellular content of mitochondria changes dynamically during development and in response to external stimuli, but the underlying mechanisms remain obscure. To systematically identify molecular probes and pathways that control mitochondrial abundance, we developed a high-throughput imaging assay that tracks both the per cell mitochondrial content and the cell size in confluent human umbilical vein endothelial cells. We screened 28,786 small molecules and observed that hundreds of small molecules are capable of increasing or decreasing the cellular content of mitochondria in a manner proportionate to cell size, revealing stereotyped control of these parameters. However, only a handful of compounds dissociate this relationship. We focus on one such compound, BRD6897, and demonstrate through secondary assays that it increases the cellular content of mitochondria as evidenced by fluorescence microscopy, mitochondrial protein content, and respiration, even after rigorous correction for cell size, cell volume, or total protein content. BRD6897 increases uncoupled respiration 1.6-fold in two different, non-dividing cell types. Based on electron microscopy, BRD6897 does not alter the percent of cytoplasmic area occupied by mitochondria, but instead, induces a striking increase in the electron density of existing mitochondria. The mechanism is independent of known transcriptional programs and is likely to be related to a blockade in the turnover of mitochondrial proteins. At present the molecular target of BRD6897 remains to be elucidated, but if identified, could reveal an important additional mechanism that governs mitochondrial biogenesis and turnover.

## Introduction

The cellular abundance of mitochondria varies across organisms, organs, and in response to environmental cues. Kleiber [1] noted that the total amount of mitochondria in a given organism, as measured by whole body respiration, scales across organisms according to a power law of the body mass. Mitochondria abound in the heart, brown fat, and skeletal muscle [2], while mature red blood cells are devoid of mitochondria. Changes in energy demand [3] and particular signaling events [4], [5] can modulate mitochondrial content. Moreover, the cellular content of mitochondria, based on electron microscopy, changes in proportion to cell size throughout the cell cycle [6].

While variation in mitochondrial content across these lengths and time scales has been documented, the underlying mechanisms remain to be fully elucidated. It has been studied most extensively at the transcriptional level. Mitochondrial content in many cell types is enhanced through a carefully studied transcriptional program involving the PGC-1 family of coactivators [7]–[9] that partner with key transcription factors ERRA [10], [11], NRF1 [12], [13], and NRF2 (GABP) [14]–[16]. With the exception of these transcriptional programs, little is known about the molecular mechanisms governing the cellular content of mitochondria.

To systematically identify molecular probes and new pathways that regulate mitochondrial content, we performed an image-based screen across 28,786 compounds. Hundreds of compounds elevated mitochondrial content in a manner proportionate to cell size. However, a few compounds, including BRD6897, were able to elevate mitochondrial content without altering cell size. BRD6897 increased the density of mitochondria and respiration independent of known transcriptional mechanisms. The screening approach and this tool compound could prove useful in discovering new pathways that control the cellular content of mitochondria.

## Results and Discussion

**Figure 1:**
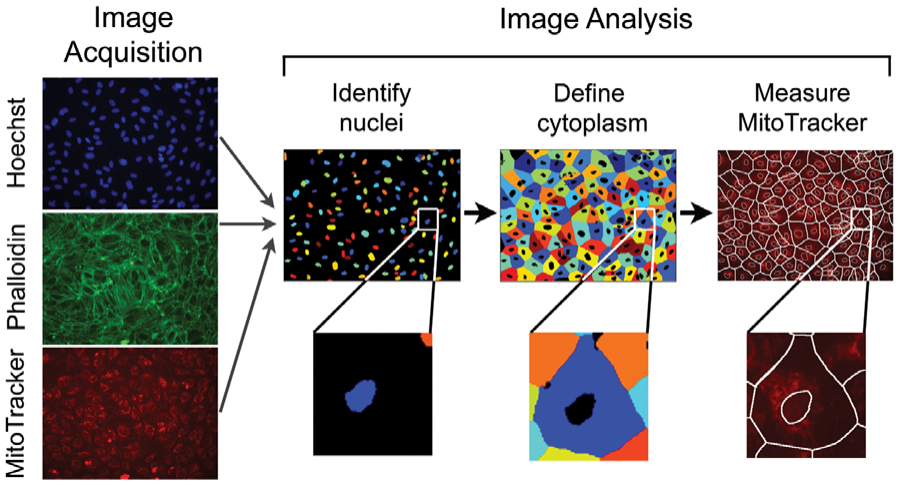
An image-based assay for mitochondrial content and cell size.

To monitor changes in cellular mitochondrial content, we developed a fluorescent image-based assay (Figure 1) in human umbilical vein endothelial cells (HUVECs). These primary human cells grow as a monolayer and have a flat morphology, which is ideal for image analysis. Hoechst nuclear stain was used to identify each nucleus in the images, the F-actin stain phalloidin was used to define the boundary of the cell, and MitoTracker Deep Red was used to monitor changes in mitochondrial content. For each cell, total MitoTracker intensity as well as that cell's cytoplasmic area were measured. For a given image, median values were calculated from the population of cells.

**Figure 2:**
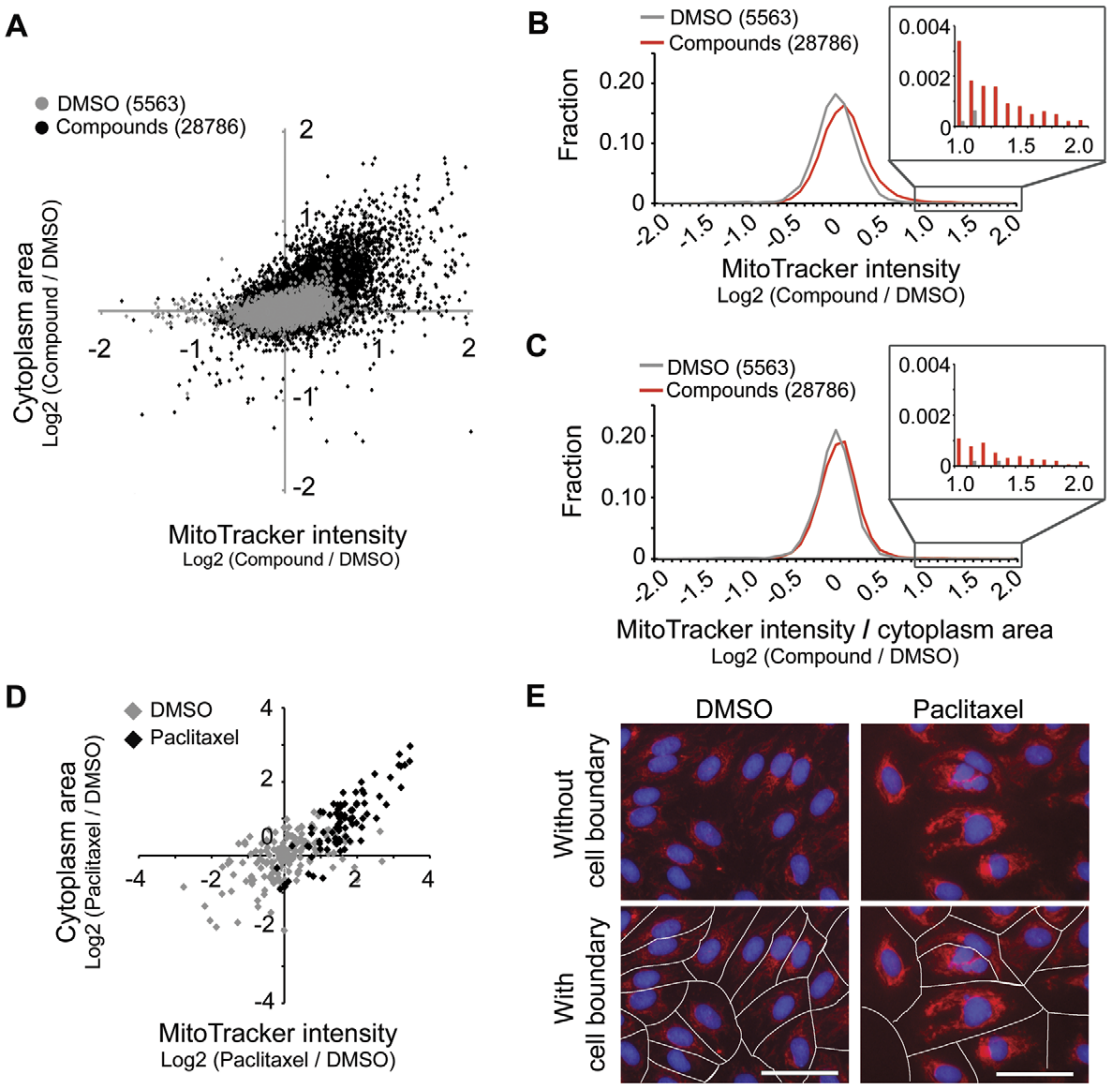
The relationship between mitochondrial content and cell size revealed through 28,786 chemical perturbations. (**A**) MitoTracker intensity per cell versus cytoplasm area per cell for each compound relative to DMSO, reported as the median value across the population of cells in an image. The median values were averaged per compound across biological duplicates and represented by a dot. (**B**) Frequency distribution of (MitoTracker intensity) per cell. (**C**) Frequency distribution of (MitoTracker intensity/cytoplasm area) per cell. (**D**) MitoTracker intensity versus cytoplasm area for 4.6 µM paclitaxel treatment. Each dot represents one cell in an image. (**E**) Images of DMSO and paclitaxel treated HUVEC stained with Hoechst (blue) and MitoTracker (red). White lines define derived cell boundaries. Scale bar: 50 µm.

We screened in biological duplicate 28,786 small molecules treated for a three-day period (Figure 2 A). Surprisingly, we found that hundreds of compounds increased MitoTracker intensity (Figure 2 B) on a per cell level. On closer inspection, however, most of these compounds also increased cell size in proportion to the fold increase in mitochondrial content (Figure 2 A). These compounds included many of the known bioactives including previously identified enhancers of mitochondrial content [17], [18] such as microtubule modulators paclitaxel (Figure 2 D, E) and deoxysappanone. The screening results support and extend the original observations of Posakony *et al.* [6] that cell size and mitochondrial content are strongly coupled and suggest that mechanisms regulating cell size also influence mitochondrial content.

Hence, we became particularly interested in molecules capable of dissociating the relationship between cell size and mitochondrial content, as they could potentially reveal novel mechanisms controlling mitochondrial content (Figure 2 C). The 160 highest scoring compounds that increased MitoTracker intensity after normalizing for cell size were selected for secondary assays. The assays consisted of immunofluorescence assays for mitochondrial protein content and qPCR assays for mitochondrial DNA (mtDNA) copy number. Ten highest scoring compounds from each secondary assay were promoted for oxygen consumption assays to ensure that elevations in mitochondrial content corresponded to an increase in functional respiratory capacity. Of the 20 compounds tested, three compounds (BRD6897, BRD6445, BRD1108) showed statistically significant increases in uncoupled respiration (Figure S1).

**Figure 3:**
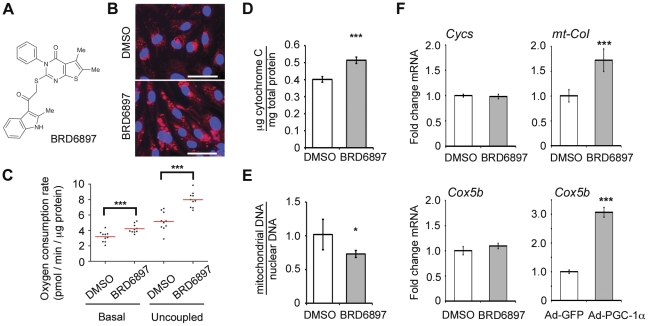
Effects of BRD6897 on mitochondrial content, gene expression, and physiology. (**A**) Chemical structure of BRD6897. (**B**) Images of DMSO- and BRD6897-treated HUVEC stained with Hoechst (blue) and MitoTracker (red). Scale bar: 50 µm. (**C**) Basal and uncoupled respiration of HUVEC treated for 3-days with 10 µM BRD6897. (**D**) Cytochrome C protein level detected by ELISA normalized to total protein content after 3-day treatment with 10 µM BRD6897. (**E**) Ratio of mitochondrial to nuclear DNA copy number after 3-day treatment with 10 µM BRD6897. (**F**) qPCR analysis of OXPHOS genes upon 3-day BRD6897 treatment. mtOXPHOS (mt-CoI) and nuclear encoded OXPHOS genes (Cycs, Cox5b) were profiled. As a positive control for activation of mitochondrial biogenesis program, PGC-1α was over-expressed for three days and compared to GFP over-expression for three days. Changes in treatment relative to control were analyzed with unpaired t-test. * p<0.05, ** p<0.01, *** p<0.001.

We chose to focus on one of these three compounds, namely BRD6897 (Figure 3 A), as it imparted the greatest, dose-dependent increase in uncoupled respiration (Figure S2). BRD6897 comes from a kinase inhibitor-biased library of compounds that are predicted to bind the ATP ligand site of kinases, though its specific target is not known. The binding ability of BRD6897 to a panel of 442 kinases [19] was tested using 10 µM compound concentration but failed to reveal any significant binding. The result suggests that BRD6897 does not have strong affinity for a range of kinases often observed with known kinase inhibitors [19], [20]. In our cells, a 10 µM dose of BRD6897 induces a 1.55 fold increase in uncoupled respiration (Figure 3 C). Additional screening of 36 structural analogs of BRD6897 not present in the initial screening collection failed to reveal more potent inducers of mitochondrial content (Figure S3). An increase in uncoupled respiration with BRD6897 is detectable by two days of treatment (Figure S4 A), plateaus after three days of treatment (Figure S4 B), and is partially reversible after removal of the compound for three days (Figure S4 C). The increase in respiration is robust to the normalization scheme used. BRD6897 increases respiration when normalized to total cell number [1.35-fold], total cell volume [1.46-fold], or total cellular protein [1.57-fold]. The compound is toxic in dividing cells but is capable of enhancing uncoupled respiration in other non-dividing cells. For example, it induces a 1.60-fold elevation in uncoupled respiration in confluent 3T3-L1 preadipocytes at a dose of 10 µM (Figure S5).

We next sought to identify the cellular pathways through which BRD6897 might be acting. If it is acting via the known transcriptional pathways of mitochondrial biogenesis [8], we would expect concomitant elevations in both nuclear DNA and mitochondrial DNA (mtDNA) encoded OXPHOS genes. In addition, mtDNA copy number itself as well as OXPHOS protein level should increase. Contrary to this expectation, BRD6897 shows no significant elevation in nuclear DNA encoded OXPHOS gene expression while mtDNA encoded OXPHOS gene expression is increased (Figure 3 F). Moreover, BRD6897 actually reduces mtDNA copy number (Figure 3 E) while elevating OXPHOS protein content (Figure 3 D). These findings indicate that BRD6897 is not acting through the canonical transcriptional programs and suggests that other mechanisms are at play.

**Figure 4:**
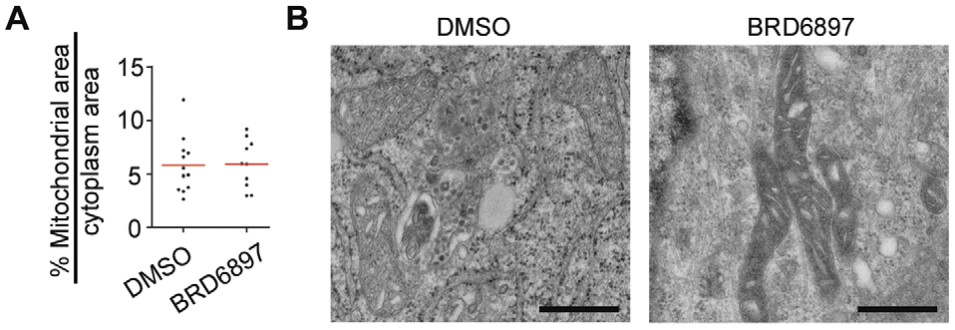
Changes in mitochondrial ultrastructure upon 3-day 10 µM BRD6897 treatment. (**A**) Plot of % cytoplasmic area occupied by mitochondria based on 10 replicate electron microscopy (EM) images per treatment. (**B**) Representative EM images of DMSO- and BRD6897-treated HUVEC. Scale bar: 500 nm.

To examine the mechanisms responsible for the increase in uncoupled respiration, we studied the morphology of mitochondria by electron microscopy. The proportion of cytoplasm area occupied by mitochondria in BRD6897-treated HUVEC did not differ significantly from DMSO-treated HUVEC (Figure 4 A). This is in contrast to the increase in mitochondrial area observed during PGC-1α mediated mitochondrial biogenesis [8]. Instead, mitochondria in BRD6897-treated HUVEC appeared more electron dense compared to mitochondria in DMSO-treated HUVEC (Figure 4 B) suggesting increased protein density within mitochondria. We next examined whether OXPHOS protein synthesis or degradation was altered upon BRD6897 treatment using pulse-chase labeling. BRD6897 did not increase the rate of cytochrome C synthesis (Figure S6, left). However, BRD6897 slowed the rate of cytochrome C degradation (Figure S6, right) suggesting that mitochondrial protein turnover is partially inhibited by BRD6897. Therefore, BRD6897 does not appear to induce mitochondrial biogenesis via transcriptional programs, but perhaps through a mechanism involving decreased mitochondrial protein turnover.

In summary we have performed a high-throughput, high-content microscopy assay that reveals a stereotyped, coordinated control of mitochondrial content and cell size preserved across hundreds of small molecule perturbations. Given that growth factors [21], [22] such as VEGF and downstream mitogenic signals such as myc [22], [23] are known to induce mitochondrial biogenesis as well as stimulate cell growth, these signals may be responsible for ensuring the strong coupling between mitochondrial content and cell size. Importantly, we identified a handful of molecules that are unique in their ability to disrupt the strong relationship between mitochondrial content and cell size. One such compound, BRD6897, increases cellular mitochondrial content on the basis of microscopy, protein content, and respiration in two different non-dividing cell types. It does not appear to activate the known transcriptional programs of mitochondrial biogenesis, but rather, appears to influence mitochondrial content perhaps by modulating protein turnover. At present the precise molecular target of BRD6897 remains to be elucidated, but if identified, could reveal an important new pathway, that together with transcriptional programs of mitochondrial biogenesis, would serve to regulate mitochondrial content.

## Materials and Methods

### Cell culture

Human umbilical vein endothelial cells (HUVEC) from a single donor was obtained from Lonza and grown in EGM2 media (Lonza). All experiments were performed between passage 3 and 6. 3T3-L1 preadipocyte was grown in DMEM with 4 mM glutamine and 1 mM sodium pyruvate supplemented with 10% calf serum and penicillin/streptomycin. All cells were grown at 37°C in 5% CO_2_.

### Compound collection

Compound libraries were assembled from the following suppliers. Bioactives: 1920 compounds from Biomol ICCB Bioactive, Neurotransmitter, and Kinase Inhibitor libraries, the MicroSource Spectrum library, and Prestwick Chemical library. Natural products: 1600 compounds from AnalytiCon's MEGx collection excluding unpurified and partially purified extracts. Commercial compounds: 3520 drug-like libraries from TimTec, Maybridge, and ChemDiv. Kinase inhibitor biased compounds: 11520 compounds from ChemBridge's KINA set. Chemical biology production libraries: 10226 compounds from diversity-oriented synthesis efforts at the Broad Institute Chemical Biology Platform. All compounds were previously tested for purity and identity using liquid chromatography-mass spectrometry (LC-MS).

### Image-based primary screen

HUVEC were seeded in 384-well CellBIND black-wall plate (Corning) at 4000 cells per well in 50 µL of EGM2 and allowed to reach confluence for two days. EGM2 was replaced and 100 nL of compounds in DMSO were pin-transferred resulting in 10–20 µM final concentration. After 3-day incubation with compounds, cells were treated with 200 nM MitoTracker Deep Red (Invitrogen) for 30 minutes at 37°C in 5% CO_2_, fixed in 4% paraformaldehyde for 20 minutes, permeabilized with 0.2% Triton-X (Sigma) for 5 minutes, and stained with 2 µg/mL Hoechst 33342 (Invitrogen) and 130 nM Alexa Fluor 488 phalloidin (Invitrogen) for 30 minutes. Cells were imaged with the ImageXpress Micro System (Molecular Devices) at 20× magnification, one site per well, with binning of 1 and gain of 2 using laser-based focusing. Images were captured using a DAPI filter (387/11 nm Ex, 447/60 nm Em) for 50 msec, Cy5 filter (628/40 nm Ex, 692/40 nm Em) for 1000 msec, and GFP filter (472/30 nm Ex, 520/35 nm Em) for 750 msec for Hoechst 33342, MitoTracker Deep Red, and AlexaFluor 488 phalloidin respectively. All compounds were screened in duplicate and each day of screening contained DMSO negative control plates.

### Image processing

All images were processed using CellProfiler [24]. Raw images were first corrected for uneven illumination per channel per plate. For MitoTracker signal, a background threshold was applied using Otsu three-class thresholding. For each well, nuclei were first identified using Hoechst stain. Then cell boundaries were identified using phalloidin stain using the propagation algorithm [25] where distances between nuclei are used to assist in identifying cell boundary. The nucleus region was masked from the whole cell to define the cytoplasm. Nucleus masking allowed us to discount background MitoTracker staining in the nucleus that could not be eliminated with background thresholding. For each cell, total MitoTracker intensity within the cytoplasm, and cytoplasm area, were measured. MitoTracker intensity was also normalized to cytoplasm area for each cell. Full detail of the image processing pipeline is available at: http://www.cellprofiler.org/published_pipelines.shtml.

### Screening data analysis

For each image, the median value of MitoTracker intensity normalized to cytoplasm area was computed. This median value was converted to composite Z-score as described previously [26]. To filter out chemicals that were toxic, we selected out wells with cell counts that were less than 40 cells per image, corresponding to about 30% of average cell count in negative control DMSO wells. Compounds with Z-score greater than 1.98 (p<0.05) were manually examined to filter out false hits that arise from staining artifacts or errors in cell segmentation. The top 160 compounds that passed filtering were run on LC-MS to ensure purity and identity of hit compounds, and re-plated into cherry pick plates for high-throughput secondary assays.

### Immunofluorescence-based secondary assay

HUVEC were treated with 2 µg/mL ATP synthase subunit alpha monoclonal antibody (MitoSciences) and 2 µg/mL AlexaFluor 647 anti-mouse IgG2b (Invitrogen) according to manufacturer's immunocytochemistry protocol. Images were analyzed as primary assay and top 10 compounds were nominated for oxygen consumption assay. Additional details are in methods S1.

### mtDNA copy number secondary assay

DNA was prepared from HUVEC using HotSHOT protocol [27], diluted in water 1∶64, and qPCR was performed using mtND2 (mtDNA encoded) and Alu (nuclear DNA encoded) probes using TaqMan reagents (Applied Biosystems). Top 10 compounds with increased mtDNA/nuDNA ratio were nominated for oxygen consumption assay. Additional details are in methods S1.

### Oxygen consumption assay

Oxygen consumption rates (OCR) were measured with XF24 Analyzer (Seahorse Biosciences) using manufacturer's protocol [28] for basal OCR followed by incubation with 1 µM carbonyl cyanide 3-chlorophenylhydrazone (CCCP) for uncoupled OCR. Basal and uncoupled OCRs per well were averaged across replicate measurements and then normalized to total protein content. Compounds that showed statistically significant increase in uncoupled respiration were re-purchased from suppliers, re-tested, and used for subsequent studies. BRD6897 (PubChem ID: 1252659, ChemBridge catalog #: 7930518), BRD6445 (PubChem ID: 2914607, ChemBridge catalog #: 6804267), BRD1108 (PubChem ID: 2213867, ChemBridge catalog #: 7852208). Additional details are in methods S1.

### Cell counting, cell volume, and total protein content measurement

Cell count and cell volume were determined by Scepter cell counter (Millipore) according to manufacturer's instruction. Total protein content was quantified with Pierce BCA Protein Assay Kit (Thermo Scientific) according to manufacturer's instruction.

### Cytochrome C ELISA

Cytochrome C from total cell lysate was detected using human cytochrome C Quantikine ELISA kit (R&D Systems) using manufacturer's instruction and normalized to total protein content.

### mtDNA copy number assay

DNA was extracted using DNeasy kit (Qiagen) and the same qPCR probes were used as the high-throughput mtDNA assay.

### Real-time PCR assay

Total RNA was extracted with RNeasy Mini kit (Qiagen) and genomic DNA was digested using RNase-Free DNase (Qiagen). 200 ng of total RNA was reverse transcribed using TaqMan reverse transcription reagents (Applied Biosystems) with random hexamers. The relative quantity of cDNA was measured using TaqMan universal PCR mastermix (Applied Biosystems) using following primer/probes: Cox5b (Hs00426948_m1), Cycs (Hs01588974_g1), mt-CoI (Hs02596864_g1), Actb (4352935).

### Transmission electron microscopy

HUVEC were prepared for electron microscopy as before [29] and imaged at 23,000×. Cytoplasm area and mitochondrial area were analyzed manually with Image J software [30]. Additional details are in methods S1.

## Supporting Information

Figure S1 **Three compounds that increase uncoupled respiration even after correcting for cell size.** HUVEC were treated for 3-days with 10 µM of BRD6897, 10 µM of BRD6445 or 20 µM of BRD1108 with DMSO as a negative control. Ten biological replicates per treatment group are represented by dots. Fold change is indicated in the graph. Differences between DMSO and compound treatment were determined by t-test. *** p<0.001.
(TIF)

Figure S2 **Impact of BRD6897 on uncoupled respiration.** HUVEC were treated for 3-days with either DMSO negative control or BRD6897 at 5 µM, 10 µM, and 20 µM concentration. Ten biological replicates per treatment group are represented by dots. Fold change is indicated in the graph. Differences between DMSO and BRD6897 were determined by t-test. ** p<0.01, *** p<0.001.
(TIFF)

Figure S3 **Screening structural analogs of BRD6897.** 36 commercially available analogs of BRD6897 were screened using the primary assay described in Figure 1. Four analogs that showed significant MitoTracker intensity normalized to cytoplasm area were followed up with respiration measurements at 5 µM and 20 µM concentration. Activities of analogs based on uncoupled respiration are described as percent of BRD6897 uncoupled respiration.
(TIFF)

Figure S4 **Time dependent effects of BRD6897 treatment on respiration.** (**A**) Changes in basal and uncoupled respiration were measured after 0-day, 1-day, 2-day, and 3-day incubation with 10 µM BRD6897. All cells were in culture for the same 3-day duration with addition of BRD6897 at appropriate time points. Five biological replicates were tested. (**B**) Changes in basal and uncoupled respiration were measured after 0-day, 3-day, and 6-day incubation with 10 µM BRD6897. All cells were in culture for the same 6-day duration with addition of BRD6897 at appropriate time points. Media were changed at day 3. Six or eight biological replicates were tested as represented by dots. (**C**) Changes in basal and uncoupled respiration were measured after 6-day DMSO incubation (untreated), 3-day DMSO incubation followed by 3-day 10 µM BRD6897 incubation (treated), or 3-day 10 µM BRD6897 incubation followed by 3-day DMSO incubation (washout). Cells were washed with media 3-times on day 3. Six or eight biological replicates were tested as represented by dots. Differences between DMSO and BRD6897 treatment groups were determined by ANOVA with Bonferroni's multiple comparison test. * p<0.05, ** p<0.01, *** p<0.001.
(TIFF)

Figure S5 **Basal and uncoupled respiration in confluent 3T3-L1 preadipocytes upon BRD6897 treatment for 3 days.** Differences between DMSO and BRD6897 treatment groups were determined by ANOVA with Bonferroni's multiple comparison test. * p<0.05, ** p<0.01, *** p<0.001.
(TIFF)

Figure S6 **Changes in mitochondrial protein turnover upon BRD6897 treatment as detected by pulse-chase labeling.** HUVEC were treated with either DMSO or 10 µM BRD6897 for 2 days. Cells were then “pulse” labeled with methionine/cysteine for 2, 4, or 6 hours (left panel). Following 6 hours of labeling, the label was “chased” for 1, 3, or 6 days (right panel). Cytochrome C from protein lysate was immunoprecipitated and run on an SDS-PAGE gel.
(TIFF)

Methods S1 Additional details for secondary assays, oxygen consumption assay, transmission electron microscopy, and pulse-chase labeling and immunoprecipitation.
(DOC)
