# Supplementary figures and images for: A Chemical Screen Probing the Relationship between Mitochondrial Content and Cell Size
Source: PLoS One. 2012 Mar 29;7(3):e33755. doi: 10.1371/journal.pone.0033755 (PMC3315575; doi:10.1371/journal.pone.0033755)

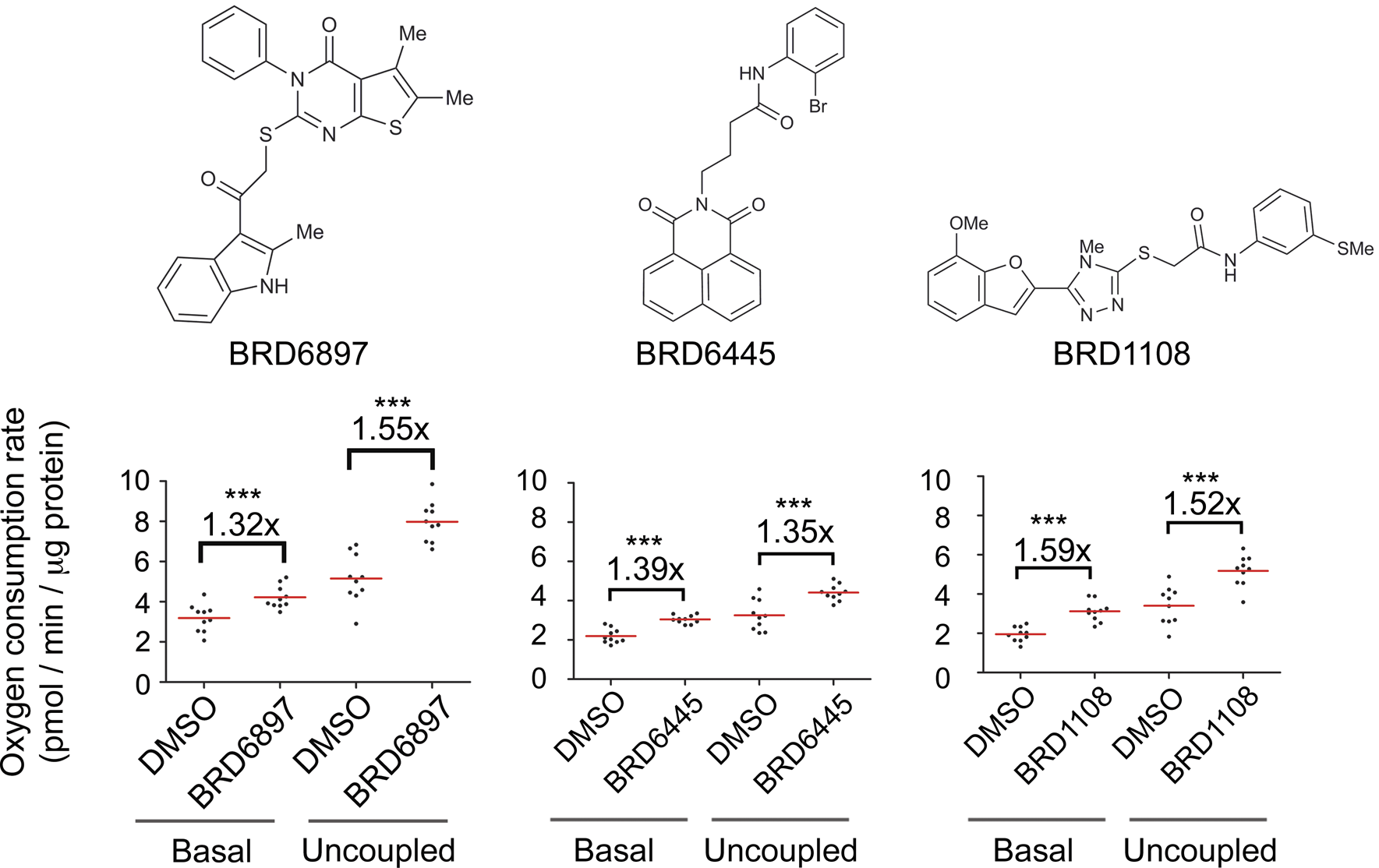

Supplement: Figure S1 — Three compounds that increase uncoupled respiration even after correcting for cell size. HUVEC were treated for 3-days with 10 µM of BRD6897, 10 µM of BRD6445 or 20 µM of BRD1108 with DMSO as a negative control. Ten biological replicates per treatment group are represented by dots. Fold change is indicated in the graph. Differences between DMSO and compound treatment were determined by t-test. *** p<0.001. (TIF) [file pone.0033755.s001.tif]

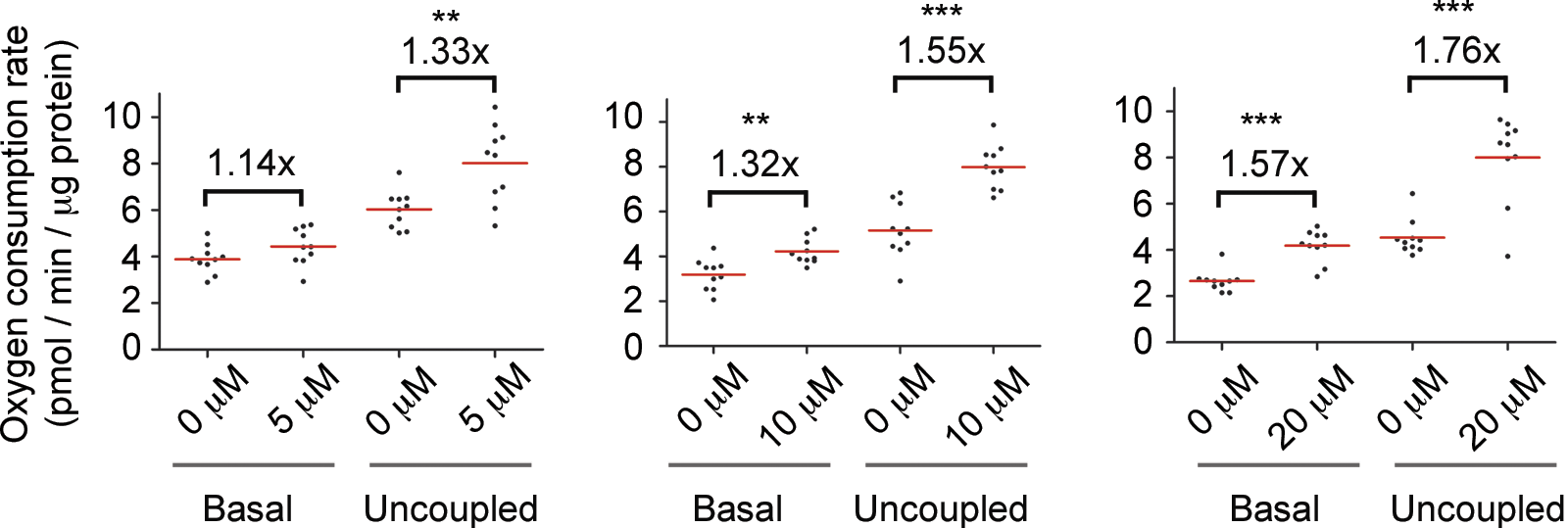

Supplement: Figure S2 — Impact of BRD6897 on uncoupled respiration. HUVEC were treated for 3-days with either DMSO negative control or BRD6897 at 5 µM, 10 µM, and 20 µM concentration. Ten biological replicates per treatment group are represented by dots. Fold change is indicated in the graph. Differences between DMSO and BRD6897 were determined by t-test. ** p<0.01, *** p<0.001. (TIFF) [file pone.0033755.s002.tiff]

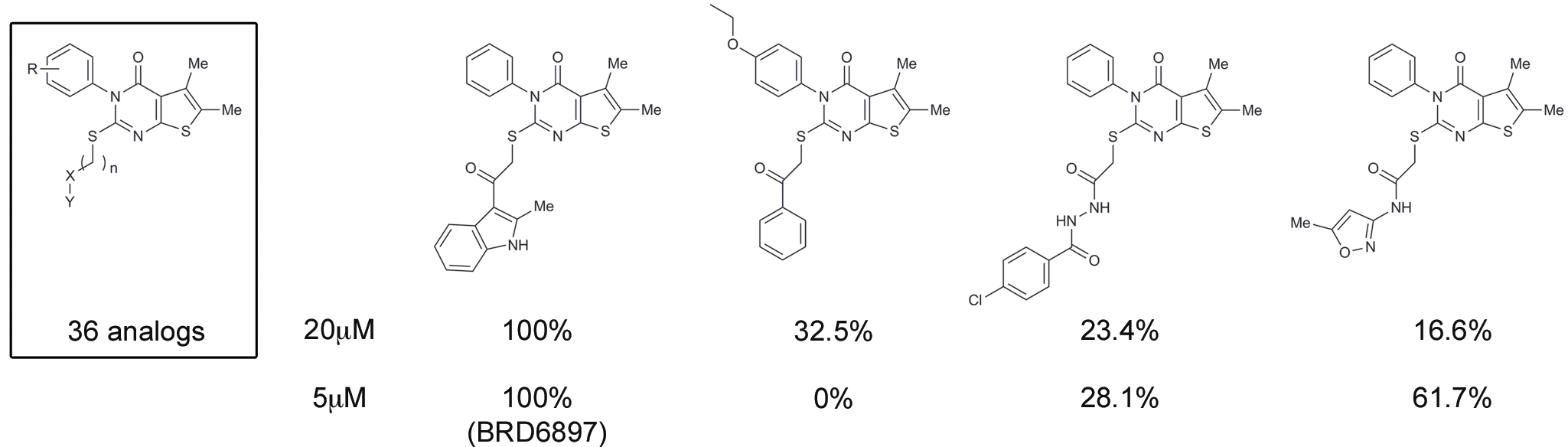

Supplement: Figure S3 — Screening structural analogs of BRD6897. 36 commercially available analogs of BRD6897 were screened using the primary assay described in Figure 1. Four analogs that showed significant MitoTracker intensity normalized to cytoplasm area were followed up with respiration measurements at 5 µM and 20 µM concentration. Activities of analogs based on uncoupled respiration are described as percent of BRD6897 uncoupled respiration. (TIFF) [file pone.0033755.s003.tiff]

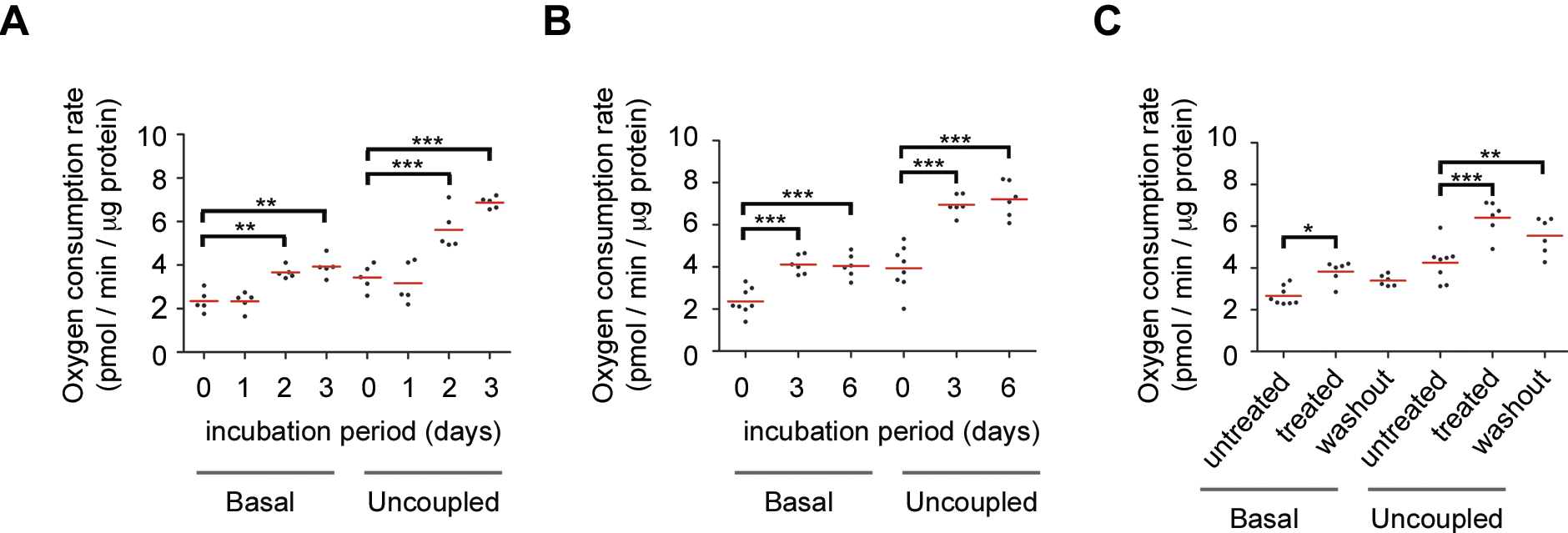

Supplement: Figure S4 — Time dependent effects of BRD6897 treatment on respiration. (A) Changes in basal and uncoupled respiration were measured after 0-day, 1-day, 2-day, and 3-day incubation with 10 µM BRD6897. All cells were in culture for the same 3-day duration with addition of BRD6897 at appropriate time points. Five biological replicates were tested. (B) Changes in basal and uncoupled respiration were measured after 0-day, 3-day, and 6-day incubation with 10 µM BRD6897. All cells were in culture for the same 6-day duration with addition of BRD6897 at appropriate time points. Media were changed at day 3. Six or eight biological replicates were tested as represented by dots. (C) Changes in basal and uncoupled respiration were measured after 6-day DMSO incubation (untreated), 3-day DMSO incubation followed by 3-day 10 µM BRD6897 incubation (treated), or 3-day 10 µM BRD6897 incubation followed by 3-day DMSO incubation (washout). Cells were washed with media 3-times on day 3. Six or eight biological replicates were tested as represented by dots. Differences between DMSO and BRD6897 treatment groups were determined by ANOVA with Bonferroni's multiple comparison test. * p<0.05, ** p<0.01, *** p<0.001. (TIFF) [file pone.0033755.s004.tiff]

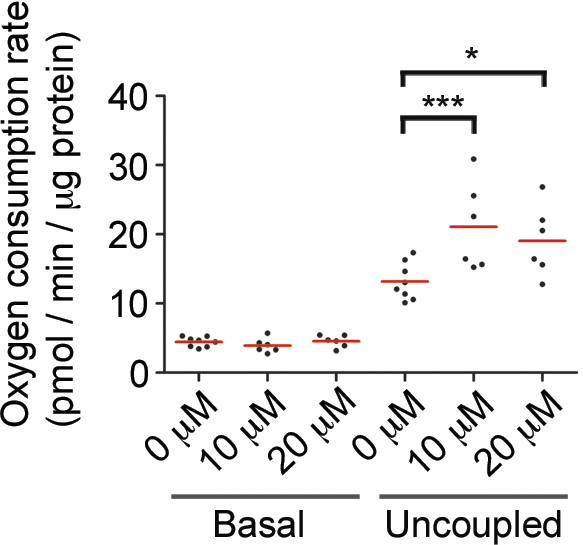

Supplement: Figure S5 — Basal and uncoupled respiration in confluent 3T3-L1 preadipocytes upon BRD6897 treatment for 3 days. Differences between DMSO and BRD6897 treatment groups were determined by ANOVA with Bonferroni's multiple comparison test. * p<0.05, ** p<0.01, *** p<0.001. (TIFF) [file pone.0033755.s005.tiff]

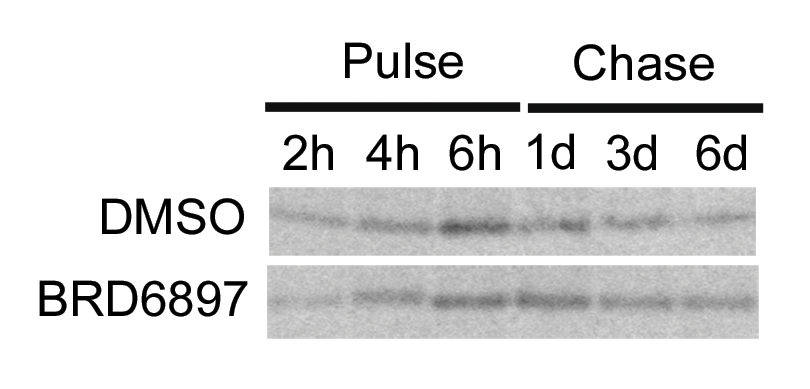

Supplement: Figure S6 — Changes in mitochondrial protein turnover upon BRD6897 treatment as detected by pulse-chase labeling. HUVEC were treated with either DMSO or 10 µM BRD6897 for 2 days. Cells were then “pulse” labeled with methionine/cysteine for 2, 4, or 6 hours (left panel). Following 6 hours of labeling, the label was “chased” for 1, 3, or 6 days (right panel). Cytochrome C from protein lysate was immunoprecipitated and run on an SDS-PAGE gel. (TIFF) [file pone.0033755.s006.tiff]
