# Supplementary material for: A Chemical Screen Probing the Relationship between Mitochondrial Content and Cell Size
Source: PLoS One. 2012 Mar 29;7(3):e33755. doi: 10.1371/journal.pone.0033755 (PMC3315575; doi:10.1371/journal.pone.0033755)
Supplement: Methods S1 — Additional details for secondary assays, oxygen consumption assay, transmission electron microscopy, and pulse-chase labeling and immunoprecipitation. (DOC) [file pone.0033755.s007.doc]

**Supplementary Methods**

**Immunofluorescence-based secondary assay.** HUVEC were fixed and permeabilized as primary assay, then blocked with 10% goat serum in PBS for 1 hour. Cells were treated with a monoclonal antibody against ATP synthase subunit alpha (MitoSciences) in 10% goat serum at 2 μg/mL overnight in 4°C, washed three times in 1% goat serum at room temperature, and treated with AlexaFluor 647 anti-mouse IgG2b (Invitrogen) at 2 μg/mL for two hours in room temperature. Cells were washed twice in 1% goat serum in PBS at room temperature and incubated with 2 μg/mL Hoechst 33342 (Invitrogen) and 130 nM Alexa Fluor 488 phalloidin (Invitrogen) for 30 minutes. Images were processed as the primary screening assay. Top 10 compounds were nominated for tertiary assay.

**mtDNA copy number secondary assay.** HUVEC were lysed in 25 mM NaOH, 0.2 mM EDTA, pH 12.3 and incubated for 5 minutes at room temperature with shaking at 2000 rpm. 20 μL of lysate were heated at 95°C for 15 minutes, cooled to 4°C, and 20 μL of neutralization buffer (40 mM Tris-HCl, pH 4.9) were added. qPCR for mtDNA were performed in 15 μL reaction using 5 μL of diluted (1:64 dilution in water) neutralized cell lysate, 1x TaqMan universal PCR mastermix (AppliedBiosystems), and mtND2 probe mix (900 nM primers, 250 nM probe) and Alu probe mix (1000 nM primers, 100 nM probe). mtND2 refers to mitochondrial DNA sequence and Alu is a human repeat sequence in the nuclear DNA. The primers and probe sequences were mtND2 (Forward: tgttggttatacccttcccgtacta, Reverse: cctgcaaagatggtagagtagatga, Probe-6FAM: ccctggcccaaccc) and Alu (Forward: cttgcagtgagccgagatt, Reverse: gagacggagtctcgctctgtc, Probe-VIC: actgcagtccgcagtccggcct).

**Oxygen consumption assay.** HUVEC were seeded in XF 24-well cell culture microplates (Seahorse Biosciences) at 8,000 cells per well in EGM2 and allowed to reach confluence for 2 days. Prior to oxygen consumption rate (OCR) measurement, cells were washed once with assay medium (Seahorse Bioscience) at 5 mM glucose and replaced with 850 μL of assay medium and incubated at 37°C for 30 minutes in atmosphere CO_2_. Three measurements of basal OCR (3 minutes each), were followed by carbonyl cyanide 3-chlorophenylhydrazone (CCCP) injection to reach 1 μM final concentration, followed by four measurements of uncoupled OCR (3 minutes each) using XF 24 Analyzer (Seahorse Biosciences). After OCR measurements, media were aspirated and 50 μL of M-PER (Thermo Scientific) lysis buffer were added, and lysates were analyzed for total protein content using Pierce BCA protein assay kit (Thermo Scientific). Basal and uncoupled OCRs per well were averaged across replicate measurements and then normalized to total protein content.

**Transmission electron microscopy.** Confluent HUVEC in 10 cm petri dish were treated with DMSO negative control or 10 μM of BRD6897 for 3 days. Cells were fixed in 2.5% glutaraldehyde, 3% paraformaldehyde, with 5% sucrose in 0.1 M sodium cacodylate buffer (pH 7.4). Cells were then postfixed in 1% OsO4 in veronal-acetate buffer, stained in block overnight with 0.5% uranyl acetate in veronal-acetate buffer (pH 6.0), dehydrated, and embedded in Spurr’s resin. Sections were cut on a Reichert Ultracut E microtome with a Diatome diamond knife at a thickness setting of 50 nm, and stained with 2% uranyl acetate, followed by 0.1% lead citrate. Samples were examined using an FEI Tecnai Spirit TEM at 60 KV and imaged with an AMT camera at 23000x. Cytoplasm area and mitochondrial area were manually analyzed with Image J software.

**Pulse-chase labeling.** Confluent HUVEC were treated with DMSO or 10 μM of BRD6897 for two days in EGM2, then washed twice with PBS, and pre-incubated in labeling media (methionine/cysteine -free RPMI1640 (Invitrogen) supplemented with dialyzed FBS and 2 mM glutamine) for 15 minutes, then replaced with labeling media containing ^35^S methionine / cysteine (MP Biomedicals) at 50 μCi/mL for 2, 4, or 6 hours. For chase experiments, following 6 hours of methionine / cysteine labeling, cells were washed twice with EGM2 (Lonza) and incubated with EGM2 for 1, 3, or 6 additional days. Cells were washed twice with ice-cold PBS and lysed in 400 μL of lysis buffer (50 mM Tris-Cl, 300 mM NaCl, 5 mM EDTA, 0.02% (w/v) sodium azide, 1% Triton-X, pH 7.4) containing 1x Halt Protease/Phosphatase inhibitor (Thermo Scientific). Lysate were scraped and centrifuged, and supernatant was stored in -20°C.

**Cytochrome C immunoprecipitation** 8 μL of anti-cytochrome C antibody (BD Biosciences, #556432) at 0.5 mg/mL concentration were conjugated to 30 μL of 50% (v/v) protein G-Sepharose 4 fast flow (GE Healthcare) for 2 hours at 4°C. ^35^S-methionine / cysteine labeled cell lysate were mixed with the conjugate overnight in 4°C. Antibody-sepharose were washed with ice-cold wash buffer (50 mM Tris-Cl, 300 mM NaCl, 5 mM EDTA, 0.02% (w/v) sodium azide, 0.1% Triton-X, pH 7.4) three times, eluted with 60 μL of 100 mM glycine (pH 2.8) and neutralized with 6 μL of 1 M Tris (pH 8.0). The eluted proteins were ran on SDS-PAGE gel, fixed in 10% galacial acetic acid / 20% methanol for 30 minutes, dried, and imaged with PhosphorImager system (Molecular Dynamics).
